# Early post-trauma wound microbiota and its association with pain outcomes and mental health in combat-related extremity injuries: a prospective analysis

**DOI:** 10.3389/fpain.2025.1564994

**Published:** 2025-04-29

**Authors:** Kateryna Ksenchyna, Dmytro Dmytriiev, Kostiantyn Volanskyi, Oleh Ksenchyn, Oleksandr Nazarchuk

**Affiliations:** National Pirogov Memorial Medical University, Vinnytsya, Ukraine

**Keywords:** limb injuries, infected wound, pain, PTSD, depression, microbiota

## Abstract

**Introduction:**

Given that many armed conflicts are currently ongoing worldwide, a thorough study of issues related to providing medical care for the wounded is essential.

**Material and methods:**

We included 45 participants aged 20–60 years with limb injuries in our study. The participants were surveyed using a visual analog pain scale, the PHQ-9, and the PTSD-5. We formed three groups: the first group included patients with limb amputations, the second group consisted of patients with limb trauma, and the third group involved patients with limb burns.

**Results:**

We found that the average pain level in Group 1 was higher, though statistical significance was not achieved (*p* > 0,05). According to the PHQ-9, all participants exhibited depressive symptoms of varying severity. In the trauma group, patients reported fewer PTSD symptoms. Among the amputees, a significant predominance of Gram-negative microorganisms was noted. The correlation between the slightly higher pain levels and the significant predominance of Gram-negative flora in amputee patients was negative (*P* > 0.05).

**Conclusions:**

In the amputee group, there was a trend toward higher mean pain scores compared to the other groups (*p* > 0,05). The same presence and distribution of depressive and PTSD symptoms were observed across all groups. Correlation analysis between pain intensity and contamination with Gram-negative bacteria did not reveal a relationship between these two variables. The study requires a larger patient sample. Gram-negative pathogens such as Klebsiella pneumoniae, Acinetobacter baumannii, Pseudomonas aeruginosa, Klebsiella oxytoca, and Proteus mirabilis were found more frequently among all patients.

## Introduction

Based on the literature, it is known that approximately 8 million people worldwide have wounds, with or without infection, among civilians (1). The prolonged war in Ukraine necessitates a more detailed study of the specifics of providing medical care to patients with injuries. The most frequently described mechanisms of injury in the military include direct combat wounds and those resulting from explosions (2).

The issue of infection control in patients after surgical treatment remains crucial. Among the injuries sustained during the war, extremity trauma is significantly predominant (3, 4). Preventing infections in patients with combat injuries has always been important. The length of wound healing and the mortality rate are directly linked to the infection factor (5, 6, 7). Phenotypic characteristics of microorganisms play a critical role in determining virulence and the inflammatory response. Studying wound microbiota enhances the understanding of the clinical tactics required to combat wound infections (8). Microbial colonization of wound surfaces occurs from both commensal microorganisms and those that enter the wound externally after injury, during transportation at the evacuation stage, and while in medical facilities. An excessive microbial load activates more Toll-like receptors, increasing pro-inflammatory cytokines, which contributes to prolonged inflammation (9, 10). The most common infectious syndromes among combat trauma patients include skin and soft tissue infections, pneumonia, bloodstream infections, osteomyelitis, and sepsis. According to publications, Gram-negative microorganisms are recognized as the main etiological factor for infectious complications among military personnel (11, 12). Gram-negative bacteria, including *Escherichia coli*, *Klebsiella pneumoniae*, *Pseudomonas aeruginosa*, and *Acinetobacter baumannii*, are frequent causes of complications (13, 14).

Predominance of contamination by Gram-negative microorganisms is an unfavorable factor for the patient, since these pathogens are often resistant to many antibiotics. And as a result, the probability of negative consequences increases (15, 16). In addition, there are known the data about a high occurrence of *E. coli*, *K. pneumonia*, *Acinetobacter* spp. and *P. aeruginosa* producing *β*-lactamase and carbapenemase enzymes. The level of increasing resistance to aminoglycosides and fluoroquinolones is also mentioned (17–19). Certainly, these factors influence the duration of wound healing, which can subsequently contribute to the development of chronic pain and a reduction in quality of life. Pain is a subjective sensation experienced by the patient, categorized by the type of injury, its duration, and the individual's psychological state. According to the literature, the commonly recognized types of pain include nociceptive pain (resulting from soft tissue damage), neuropathic pain, and psychogenic pain. While the underlying cause of pain is typically physical, the presence of depression, stress, or anxiety can exacerbate and prolong the sensation of pain (20, 21).

An important aspect of working with patients who were wounded during military operations is the assessment of their psychological state. The specifics of military service, namely the constant stress factor and the risk of death, are a direct cause of the depression and post-traumatic syndrome development (22, 23). The prevalence of post-traumatic stress disorder according to research is 6%–8% among the general population, but among risk groups it can reach 25%. These are groups such as: war veterans, refugees and persons who have experienced violence. In addition, about 30%–40% of the risk of post-traumatic stress disorder is caused by heredity and previous injuries (24, 25).

The main aim was to study character of the wound microbiota in patients with different types of extremity injuries whose wound healing process lasted longer than one month and to analyze relations between pain intensity mental status and quality of life.

## Material and methods

### Patients

In our study, we included 45 people with limb injuries. All of them were treated at an inpatient clinic. The participants were surveyed using a visual analog pain scale (0–10 points) and the PHQ-9 questionnaire to assess the relationship between pain intensity and the presence of depressive disorders. The criteria for inclusion in the study were the presence of limb injuries: amputations, burns, and other injuries, in the period 4–12 weeks after surgical treatment, and age from 20 to 60 years. Exclusion criteria for this study were decompensated concomitant diseases, immunosuppressive conditions, cognitive disorders, and polytrauma.

During the research, we formed three groups: the 1st group was patients with limb amputations, the 2nd group was patients with limb trauma, and the 3rd group was patients with limb burns. All respondents were males, from 21 to 60 years old; statistically, the groups did not differ in age (*p* > 0,05). (Table 1).

**Table 1:** Age characteristics of the groups.

Respondents	Number of cases	Average age	Standart deviation	Normality, D'Agostino Pearson test
Group 1	18	35,77	±8,83	0,7
Group 2	12	32,75	±8,34	0,8
Group 3	15	35,73	±10,85	0,11

### VAS pain

Patient self-report is the most accurate and reliable measure of pain intensity, regardless of the patient`s age. The numerical rating scale (NRS), visual analog scale (VAS), facial pain scale (FPS), and verbal descriptor scale (VDS) have proven effective (26). We used a visual analog pain scale in all study participants to determine the novelty and intensity of pain. It is worth noting that in the group of patients with limb amputations, we evaluated only residual pain, the severity of phantom limb pain was not taken into account.

### Mental status аnalysis

All respondents answered the questions of Patient Health Questionnaire-9 (PHQ-9) and Posttraumatic Stress Disorder (PTSD-5).

PHQ-9 questionnaire is used for screening the presence and severity of depressive symptoms during the past two weeks. The questionnaire consists of nine obligatory and one additional question if the patient gets 3 points according to his answers, which are rationing such as “did not bother at all” 0 points, “several days” 1 point, “more than half of all days” 2 points, “almost every day” 3 points. A higher score indicates a higher level of depressive symptoms. If the respondent receives a “cutoff score” of 10 or more points this is the basis for referral for further diagnosis of depression (27, 28).

PTSD-5 is a tool for initial assessment of the possible presence of post-traumatic stress disorder, a kind of screening method. The scale consists of 5 questions with a maximal score of 5 points. The cut-off of the patient's points for further investigations is 4 points (29).

### Microbiological study

We analyzed the microbiological characteristics of wounds in patients with various types of combat extremity injuries: burns, amputations, and trauma. All patients were treated at an inpatient clinic. The study involved 35 participants. Microbiological samples were collected from wound surfaces during the active purulent-inflammation process. All microbial isolates underwent species identification through standard microbiological testing using a traditional assay. Soft tissue samples from wound surfaces of the extremities were collected for further microbiological examination in patients whose wound healing process lasted longer than one month. The material for the study was collected from the wound surface in compliance with the necessary sanitary and hygienic requirements (hand treatment, availability of personal protective equipment, replacement of gloves after removing the bandage). Before collecting the material, the wound was washed with sterile saline and surface contamination was removed. Direct collection was carried out with a swab of viable tissue with an area of 1 cm^2^, followed by placing the sample in a previously opened container for transportation with a completed appropriate form. In document we indicated the patient's data, time, date and place of material collection, data on antibiotic therapy and time of sending to the University Microbiology Laboratory of the Pirogov National Medical University, Vinnytsya.

### Statistical analysis

In our study for statistical analysis, we used the standardized program MedCalc®. Before performing statistical calculations, we determined the normality of the data distribution using D'Agostino-Pearsons test. For normally distributed data, we used parametric statistical methods: arithmetic mean, standard deviation, Pearson`s correlation coefficient, *t*-test. In cases of non-normal distribution, the median (Me), interquartile range (IQR), and significance test (p) were calculated. The presence of differences between the studied indicators was assessed using the Mann–Whitney *U*-test. The reliability of the difference (p) was considered statistically significant at *p* ≤ 0.05.

### Bioethical commission

The Study protocols were approved by the Committee on Bioethics, National Pirogov Memorial Medical University, Vinnytsya, Ukraine (Protocol No 7, 27.05.2024).

## Results

We surveyed 45 patients on the severity of pain using VAS (Table 2).

**Table 2:** Data about the presence of pain according to VAS in all study participants.

VAS, points	Group 1 (*n*)	Group 2 (*n*)	Group 3 (*n*)
0	1	1	2
10–30	5	4	5
40–60	10	7	8
70–90	2	0	0
100	0	0	0
Normality, D'Agostino Pearson test	0,63	0,75	0,64

**Figure 1:**
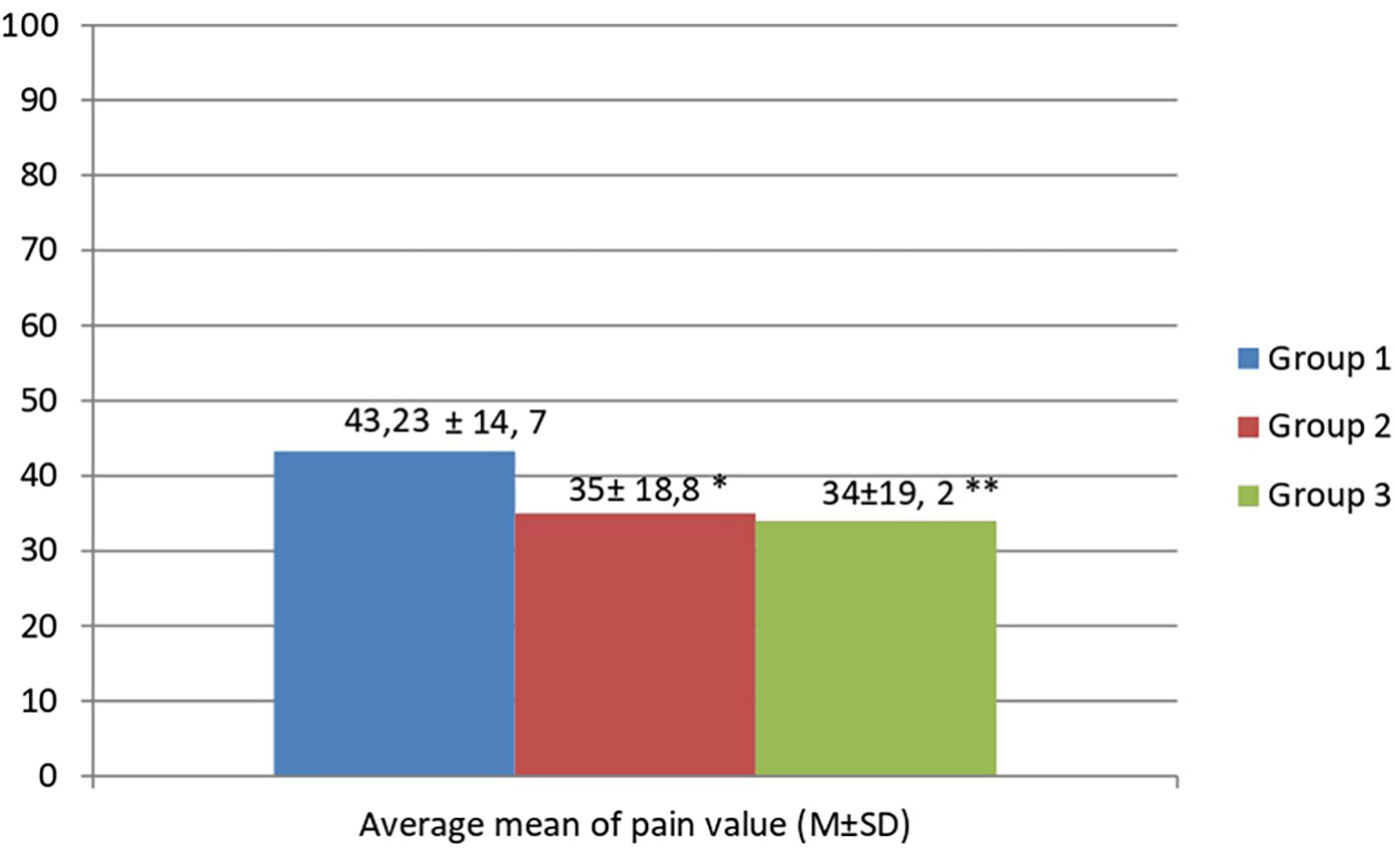
Analysis of average mean of pain value according to visual analog scale data in patient with studied groups. *p > 0.05 – When comparing the average mean pain values in groups 2 and 1, statistical significance was not established. **p > 0.05 – When comparing the average mean pain values in groups 3 and 1, statistical significance was not established.

According to the results, the average value of pain level in groups 2 and 3 did not differ significantly, while in the group of patients with amputations there was a trend toward higher mean pain scores than in the other groups, although statistical significance was not achieved (*p* > 0,05) (Figure 1).

The next stage of our study was to assess the patient's psychological status in order to investigate the interdependence of the level of pain and the level of severity of depressive symptoms. According to the PHQ-9, all participants of the study had depressive symptoms of different severity (Table 3).

**Table 3:** Received survey results PHQ-9.

Level of depressive symptoms	Group 1, % of cases	Group 2, % of cases	Group 3, % of cases
Minimal	65	46,7	58,4
Mild	25	40	33,3
Moderate	5	13,3	8,3
Medium severity	5	0	0
Severe	0	0	0

Another scale for screening PTSD also was used in the same time. According to the results, in the group with trauma, patients indicated less symptoms of PTSD (Table 4).

**Table 4:** Received survey results PTSD-5.

PTSD-5 points	Group 1, *n* (%) of cases	Group 2, (%) of cases	Group 3, (%) of cases
"0"	2 (11,1%)	5 (41,7%)	1 (6,7%)
"1–3"	13 (72,2%)	6 (50,0%)	12 (80%)
"4–5"	3 (16,7%)	1 (8,3%)	2 (13,3%)

**Figure 2:**
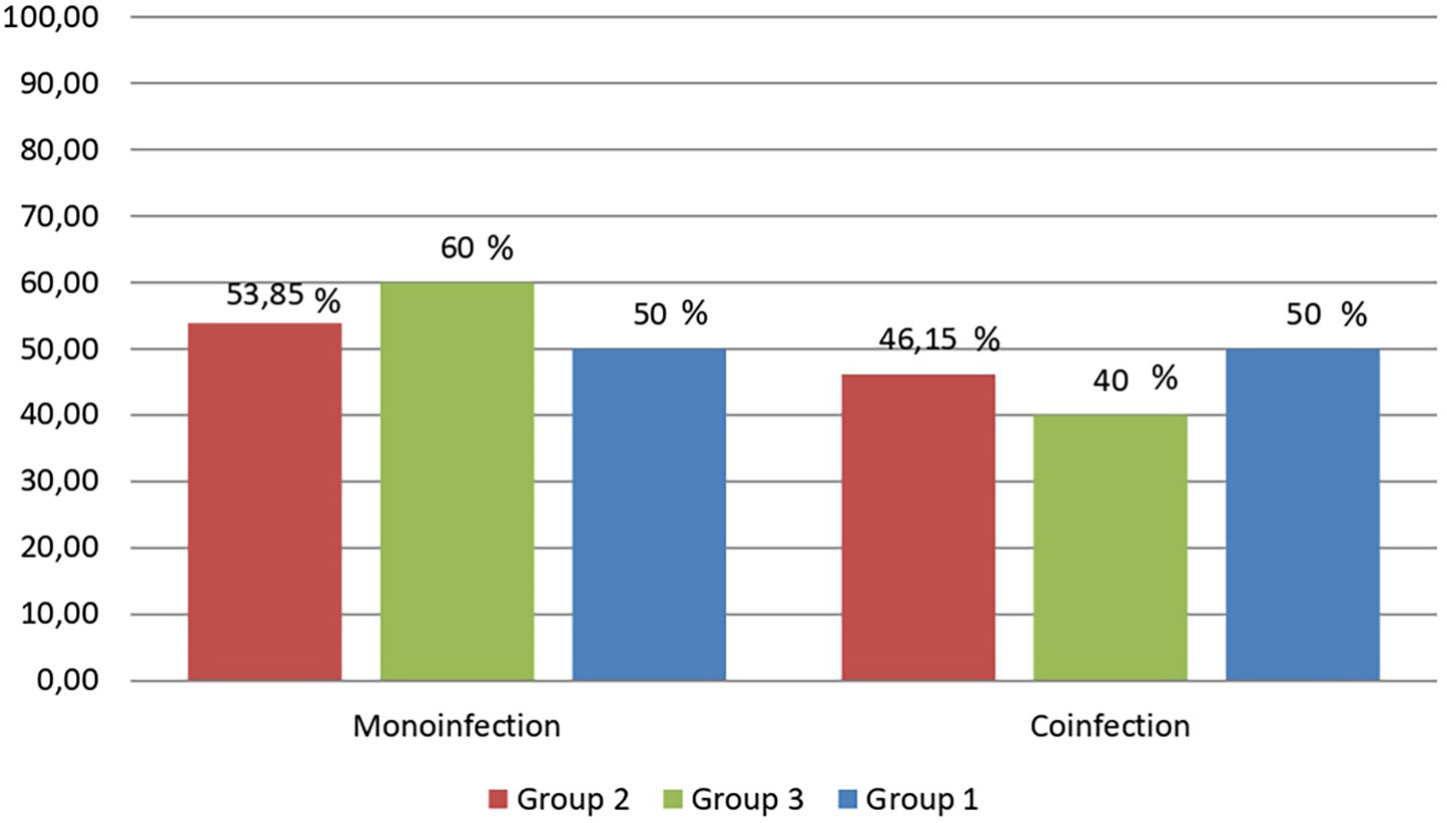
The frequency of monoinfection and combined in respondents limb wounds.

In each studied group, three subgroups were formed based on the study of the microbiological characteristics of the wound contents: 1.1 group consisted of 10 patients with amputations; 2.1 group – 12 patients with trauma; 3.1 group – 13 patients with burns. In all groups one type of microorganisms or two and more at the same time were determined equally often (Figure 2).

**Figure 3:**
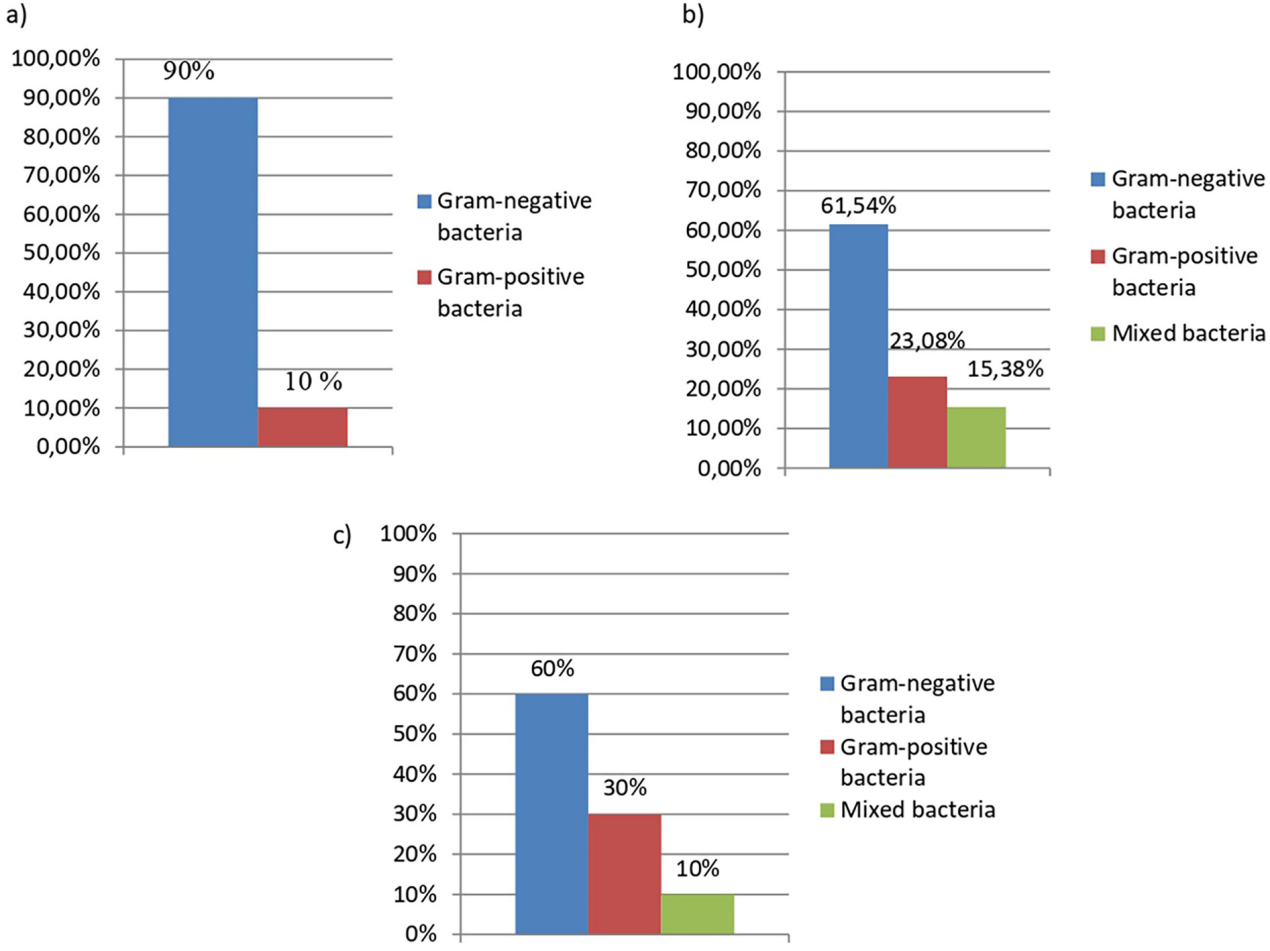
Characteristic of wound bacteria in comparison groups of patients with limb injuries. **(a)** Distribution of microorganism's detection by phenotypic characteristics as the type of their cell wall (by Gram-stainig) in patients (group 1.1, *n* = 10) with amputations. **(b)** Distribution of microorganism's detection by phenotypic characteristics as the type of their cell wall (by Gram-stainig) in patients (group 2.1, *n* = 12) with trauma. **(c)** Distribution of microorganism's detection by phenotypic characteristics as the type of their cell wall (by Gram-stainig) in patients (group 3.1, *n* = 13) with bums.

Also, one of the characteristics of microorganisms that should be paid attention to is the peculiarities of the structure of the cell wall and their division into gram-negative and gram-positive species. So in patients with amputations, a significant predominance of gram-negative microorganisms was established. In groups 2 and 3, the detection frequency of gram-negative and gram-positive bacteria and their combination were established with approximately the same distribution (Figure 3).

A detailed analysis of microbiological characteristics of extremity wounds was also carried out to identify the most common pathogens contaminating the wound in patients with extremity injuries Based on the typical distribution of bacteria, we noticed a predominance of Gram-negative microorganisms, namely *Klebsiella pneumoniae*, *Pseudomonas aeruginosa* in all groups (Table 5).

**Table 5:** The species wound characteristics in the studied groups: amputations, burns and trauma (number of cases).

Detected bacteria in wounds	Group 1	Group 2	Group 3
*Klebsiella pneumoniae + Pseudomonas aeruginosa*	2	1	1
*Klebsiella oxytoca + Pseudomonas aeruginosa*	1	0	0
*Escherichia coli + Pseudomonas aeruginosa*	1	0	0
*Enterococcus faecalis + Proteus mirabilis*	1	1	0
*Enterococcus faecalis + Pseudomonas aeruginosa*	0	0	1
*Proteus mirabilis + Pseudomonas aeruginosa*	0	0	1
*Pseudomonas aeruginosa + Staphylococcus aureus*	0	0	1
*Staphylococcus aureus + Acinobacter baumanii*	0	0	1
*Klebsiella pneumoniae + Acinobacter baumanii*	0	0	1
*Klebsiella pneumoniae + Staphylococcus aureus*	0	1	0
*Enterococcus faecalis + Acinobacter baumanii*	0	1	0
*Klebsiella pneumoniae*	1	2	2
*Klebsiella oxytoca*	1	0	0
*Acinobacter baumanii*	0	2	2
*Proteus mirabilis*	1	0	0
*Enterobacter cloacae*	1	0	0
*Escherichia coli*	1	0	0
*Staphylococcus aureus*	0	0	1
*Staphylococcus* spp.	0	0	1
*Staphylococcus epid*	0	1	0
*Pseudomonas aeruginosa*	0	1	0
*Corynobacter* spp.	0	0	1
Total	10	10	13

Taking into account the recent results, we conducted an analysis of the correlation between a slightly higher level of pain and a significant predominance of Gram-negative flora in patients with amputations. Based on the analysis, the value of the correlation coefficient −0.1225 (*P* > 0.05, 95% CI −0.7–0.55) was obtained.

Since we found a negative correlation between pain and the prevalence of Gram-negative bacteria in the wound microbiome, we calculated the statistical power of the study, which for our sample was 40%.

## Discussion

Most published research focuses on the practical aspects of treating chronic wounds and their associated pain. However, investigating the relationship between the mechanisms of chronic pain development and wound healing is an important but less-studied topic. To better understand the necessary changes in medical care provision for the wounded and to enhance rehabilitation effectiveness, it is essential to study the features of wound healing and recovery in combat conditions from various perspectives. Pain associated with chronic wounds has been examined (30), concentrating on its causes and mechanisms, including the endocannabinoid system. We compared the severity of pain and the potential for psychological disorders, noting the composition and nature of microorganisms contaminating the wound surfaces in patients with amputations, burns, and limb injuries. Thus, according to the study's results, we observed a trend toward increased pain severity in patients with amputations. However, this finding did not reach statistical significance (*p* > 0.05) compared to the other groups studied. While a difference was observed, it is not statistically compelling. Possible explanations for this include insufficient sample size and data variability. The limitations of the study are further indicated by the calculated statistical power of 40%. This value confirms the need for a larger number of participants to observe the true relationship between the level of pain perception and the presence of gram-negative bacteria in the wound microbiome. According to the literature, the power of the study is considered sufficient in the case of 80%–90%. The assessment of psychological stages using the PTSD- 5 and PHQ- 9 questionnaires revealed similar patterns of depression and PTSD symptoms across the groups of patients with amputations, burns, and other injuries. According to the survey results, patients in the trauma group scored lower for PTSD symptoms. In their study, Alexandra Florinda Ghițan et al. (2023) analyzed the relationship between trauma type and PTSD development, concluding that trauma type is crucial, as individuals with amputations and fractures are more likely to develop PTSD. Our analysis also indicated a trend where the type of trauma influenced the presence of PTSD symptoms (31). Furthermore, we discovered that in the group of patients with amputations, gram- negative microorganisms predominated, in comparison to those with burns and traumas, according to microbiological study results. Similar findings were reported by Matthew A. Soderstrom et al. (2023), who noted a higher prevalence of gram- negative organisms, such as *E. coli*, *Enterobacter* spp., and *A. baumannii* (32). Gram-negative bacteria often exhibit greater resistance to antibiotics, potentially leading to more severe infections, chronic wound development, prolonged rehabilitation periods, and a consequent decline in quality of life. The wound infection microbiome in the early period following trauma may dictate a specific microbial community structure and also influence time- dependent community dynamics.

Discussing the factors that influence pain intensity when analyzing different groups based on wound microbiota composition is challenging due to the number of variables that can influence the results. For example, the time elapsed since injury is one such factor: over time, pain may decrease due to natural healing processes. However, if the wound is infected, pain may remain severe for a long time. In addition, the use of analgesics can affect the perception of pain, as these drugs can mask or change its intensity. Equally important is the effect of antimicrobial drugs, which can change the composition of the microbiota. This makes it difficult to determine their effect on pain, after which these drugs can contribute to its reduction. The task of comparing pain levels across groups based on microbiota composition requires careful control of these factors to consider the impact of the microbiota in isolation. One possible approach is to use multivariate analysis, in which each of these variables is considered as a potential cofactor. This will avoid false conclusions and better understand the real impact of microbiota composition on pain perception.

Summarizing the above, сorrelation analysis of the level of pain and the presence of contamination with Gram-negative bacteria did not reveal a relationship between these two variables (*P* > 0.05). The study needs a larger sample of patients. Gram-negative pathogens *Klebsiella pneumoniae*, *Acinobacter baumanii*, *Pseudomonas aeruginosa*, *Klebsiella oxytoca*, and *Proteus mirabilis* were found more frequently in all patients. Since patients with amputations are predominantly colonized by gram-negative organisms capable of forming biofilms, it is worth considering the use of antiseptics and antibiotics that have activity against gram-negative bacteria in the early period after amputation to reduce the risk of chronic infections. It is mandatory to determine the sensitivity of pathogens to antibacterial drugs, which will effectively prevent their entry into the wound.

## Data Availability

The datasets presented in this study can be found in online repositories. The names of the repository/repositories and accession number(s) can be found in the article/Supplementary Material.
